# Compulsory Vaccination

**Published:** 1892-06-25

**Authors:** 


### COMPULSORY VACCINATION.

Whilst the tendency in England is towards the abolition of compulsory vaccination, in Italy and Japan it appears to be in a diametrically opposite direction. In the latter country great efforts are being made in order to prevent the spread of small-pox, and in at least one Prefecture all people under 40 years of age must be vaccinated, and must submit to a second operation if the first does not take. In Italy, in accordance with the new law, which has a stringency that would speedily arouse a bitter opposition in this country, which has always looked with jealous eyes and unmixed feelings of dislike upon compulsory legislation of any kind, every child must be vaccinated before it is six months old, and at any subsequent period of its life which may recommend itself as desirable to the sanitary authority. Those who do not submit to this law are punished by being boycotted from schools, factories, workshops, and other public institutions. Exclusion from school may commend itself to Italian youth as a highly desirable form of penalty, but being shut out from factories and workshops will probably prove a very effective punishment to the adult population.

